# TGF-β2 Induces Ribosome Activity, Alters Ribosome Composition and Inhibits IRES-Mediated Translation in Chondrocytes

**DOI:** 10.3390/ijms25095031

**Published:** 2024-05-05

**Authors:** Guus G. H. van den Akker, Alzbeta Chabronova, Bas A. C. Housmans, Laura van der Vloet, Don A. M. Surtel, Andy Cremers, Virginie Marchand, Yuri Motorin, Marjolein M. J. Caron, Mandy J. Peffers, Tim J. M. Welting

**Affiliations:** 1 Laboratory of Experimental Orthopedics, Department of Orthopedic Surgery, Research School CAPHRI, Faculty of Healthy Medicine and Life Sciences, Maastricht University, 6229 ER Maastricht, The Netherlands; a.chabronova@liverpool.ac.uk (A.C.); marjolein.caron@maastrichtuniversity.nl (M.M.J.C.); t.welting@maastrichtuniversity.nl (T.J.M.W.); 2 Department of Musculoskeletal Ageing Science, Institute of Life Course and Medical Sciences, University of Liverpool, Liverpool L7 8TX, UK; 3 UAR2008 IBSLor CNRS-INSERM, Université de Lorraine, BioPole, F54000 Nancy, France; virginie.marchand@univ-lorraine.fr (V.M.); yuri.motorin@univ-lorraine.fr (Y.M.); 4 UMR7365 IMoPA, CNRS, Université de Lorraine, BioPole, F54000 Nancy, France; 5 Laboratory of Experimental Orthopedics, Department of Orthopedic Surgery, Maastricht University Medical Center +, 6229 HX Maastricht, The Netherlands

**Keywords:** ribosome heterogeneity, differential rRNA modification, variation ribosome composition

## Abstract

Alterations in cell fate are often attributed to (epigenetic) regulation of gene expression. An emerging paradigm focuses on specialized ribosomes within a cell. However, little evidence exists for the dynamic regulation of ribosome composition and function. Here, we stimulated a chondrocytic cell line with transforming growth factor beta (TGF-β2) and mapped changes in ribosome function, composition and ribosomal RNA (rRNA) epitranscriptomics. 35S Met/Cys incorporation was used to evaluate ribosome activity. Dual luciferase reporter assays were used to assess ribosomal modus. Ribosomal RNA expression and processing were determined by RT-qPCR, while RiboMethSeq and HydraPsiSeq were used to determine rRNA modification profiles. Label-free protein quantification of total cell lysates, isolated ribosomes and secreted proteins was done by LC-MS/MS. A three-day TGF-β2 stimulation induced total protein synthesis in SW1353 chondrocytic cells and human articular chondrocytes. Specifically, TGF-β2 induced cap-mediated protein synthesis, while IRES-mediated translation was not (P53 IRES) or little affected (CrPv IGR and HCV IRES). Three rRNA post-transcriptional modifications (PTMs) were affected by TGF-β2 stimulation (18S-Gm1447 downregulated, 18S-ψ1177 and 28S-ψ4598 upregulated). Proteomic analysis of isolated ribosomes revealed increased interaction with eIF2 and tRNA ligases and decreased association of eIF4A3 and heterogeneous nuclear ribonucleoprotein (HNRNP)s. In addition, thirteen core ribosomal proteins were more present in ribosomes from TGF-β2 stimulated cells, albeit with a modest fold change. A prolonged stimulation of chondrocytic cells with TGF-β2 induced ribosome activity and changed the mode of translation. These functional changes could be coupled to alterations in accessory proteins in the ribosomal proteome.

## 1. Introduction

Osteoarthritis (OA) is an invalidating synovial joint disease. In the past few decades, the paradigm of OA as a wear-and-tear disease has changed towards that of an active disease process [1]. In addition, lifestyle factors contribute strongly to OA incidence irrespective of age [2]. This suggests that biological processes in the synovial joints are crucial for OA pathobiology and represent a treatment target. The complexity and late age onset of OA have hampered the development of successful biological treatment modalities.

The translation of mRNA into protein is carried out by the ribosome, yet ribosome function is not often studied in cartilage or OA [3]. A human cell contains ~107 ribosomes, and they take up to 4–6% of the cellular total protein mass [4]. Ribosome biogenesis takes up a large amount of the available cellular energy and is directly coupled to cell growth and proliferation. An increase in global protein synthesis, mediated by inactivation of a cap-dependent translation inhibitor 4E-BP1, was detected in a rat model of OA [5]. Interestingly, the increase in ribosome activity preceded signs of articular cartilage degeneration. TGF-β1 stimulation induced protein translation in human chondrocytes in vitro, and this was linked to AKT/mTOR-mediated inactivation of 4E-BP1 [6]. In an in vitro model for growth plate chondrogenesis, we found that the master chondrogenic regulator Sox9 increased ribosome biogenesis and activity [7,8]. Loss of TGF-β receptor signaling through Tgfbr1 leads to lethal chondrodysplasia in mice [9]. Interestingly, aging correlates with a decrease in ribosome biogenesis by epigenetic inactivation of rDNA loci [10]. Longevity studies have indicated that environmental factors that reduce ribosome biogenesis extend lifespan [11]. Taken together, these findings indicate that ribosome biogenesis and activity are tightly regulated in cartilage development and aging. Several studies indicate that changes in ribosome activity play a role in OA pathobiology [3]. Uncoupling of mRNA transcription and translation was reported in ex vivo porcine cartilage explants, where cyclic loading led to a general shutdown of protein synthesis, while chondrocyte gene expression was unaffected [12]. Joint immobilization, which reduces protein synthesis, protects against DMM-induced murine OA [13]. In addition, mechanical loading induced TGF-β signaling, and this was correlated to a reduction in Col10a1 gene expression in bovine metacarpophalangeal joint cartilage [14]. Of note, the extent of TGF-β signal activation, as determined by pSMAD2C immunohistochemistry, was reduced in aged cartilage [15]. Unloading in vivo resulted in a transition of articular cartilage to bone in rats that were immobilized for four weeks [16]. This indicates that the biomechanical activation of protein synthesis and TGF-β signaling is indeed required for articular cartilage homeostasis. Collectively, these data demonstrate that mRNA and protein expression do not correlate well in the physiological circadian loading of articular cartilage and that TGF-β can form signaling intermediates that regulate ribosome activity.

Protein translation can occur through two modes: cap- or internal ribosomal entry site (IRES)-mediated translation. The AKT/mTOR signaling primarily regulates cap-mediated translation, while stress signaling can activate IRES-mediated translation [17]. Stressors that are known to activate IRES-mediated translation are nutrient deprivation, DNA damage or inflammatory cytokines (viral infection). Many stress response genes, such as P53, p27kip, c-Myc, HIF1α, BAG1 and Bip, contain IRES elements [18]. Since OA is a disease characterized by chronic inflammation [19], occurrence of cell death [20] as well as changes in ribosome activity, ribosome modus might play a role in its (patho)biology [3]. Moreover, mounting evidence indicates that ribosomes exist in heterogeneous pools in one cell or between different cell phenotypes [21,22,23,24]. Importantly, ribosome heterogeneity has been linked to ribosome functional specialization [21,25,26]. Variations in ribosome protein composition and rRNA post-transcriptional modifications (PTMs) have been postulated as major sources of ribosome heterogeneity [21,25]. For example, ribosomes containing the ribosomal protein L10A (RPL10A) were recently shown to be upregulated and required for mesoderm specification [27]. The two most prevalent rRNA PTMs are 2′-O-methylation (2′-O-me) and pseudouridylation (Ψ). They stabilize rRNA-protein interactions and facilitate ribosome biogenesis. Previous rRNA PTM analyses demonstrated that not all positions are fully modified [28,29] and are therefore a source of ribosome heterogeneity [30].

Given the importance of TGF-β in cartilage homeostasis and disease, the uncoupling between protein translation and mRNA transcription in cartilage tissue and the observation that TGF-β can modulate ribosome activity, we aimed to test the hypothesis that TGF-β signaling not only affects global protein translation but also alters ribosome modus and composition.

## 2. Results

### 2.1. TGF-β2 Stimulation Induces Protein Translation in Chondrocytes

**Figure 1:**
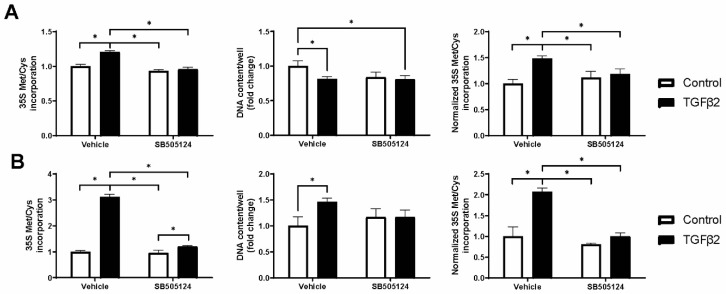
TGF-β2 stimulation enhances total protein translation. Cells were treated for 3 days with 10 ng/mL TGF-β2 or medium control in the presence of SB505124 (10 µM) or vehicle control (DMSO, Control). (**A**) Total protein translation in SW1353 chondrocytic cells and (**B**) human articular chondrocytes stimulated with TGF-β2. ^35^S-methionine/cysteine incorporation assay (left panel), DNA content (middle panel) and normalized ^35^S-methionine/cysteine incorporation (right panel). Normalization of ^35^S-methionine/cysteine incorporation (left panel) was done by correcting for the total DNA content using the data from the Sybr Green DNA assay (middle panel). Bar graphs show mean ± standard deviation (n = 3). Statistical analysis was performed with two-way ANOVA and multiple comparisons between all conditions. Statistically significant changes are indicated by asterisks (<0.05).

To evaluate the effect of TGF-β2 on total protein translation, we exposed SW1353 cells and primary human articular chondrocytes (HACs) to 35S-methionine/cysteine in the presence or absence of TGF-β2. Three days of TGF-β2 stimulation enhanced ribosome activity by 1.5-fold in SW1353 cells without inducing cell proliferation (Figure 1A). In HACs, the effect of TGF-β2 on protein translation was larger (2.1-fold), and this was accompanied by an increase in cell number (Figure 1B). TGF-β binds to the transforming growth factor receptor 1 (TGFBR1) at the cell membrane, which in turn activates downstream signaling [31]. The TGFBR1 kinase inhibitor SB505124 was used to test if the effect on protein translation was specific to TGF-β2 signaling. The inhibitor abolished the effect of TGF-β2 on cell number and protein synthesis (Figure 1A,B). Labeling with a non-radioactive methionine analogue confirmed these observations in SW1353 and primary HACs (Supplementary Figure S1). Although mTOR signaling was previously shown to be modulated by TGF-β2 in HACs [6], we did not find an effect on phosphorylation of mTORSer2448 or 4E-BP1Thr37/46 in our experimental setup in SW1353 (Supplementary Figure S2). Overall, prolonged stimulation with TGF-β2 induced protein translation in SW1353 and HACs.

### 2.2. TGF-β2 Promotes Cap-Mediated Translation in Chondrocytes

**Figure 2:**
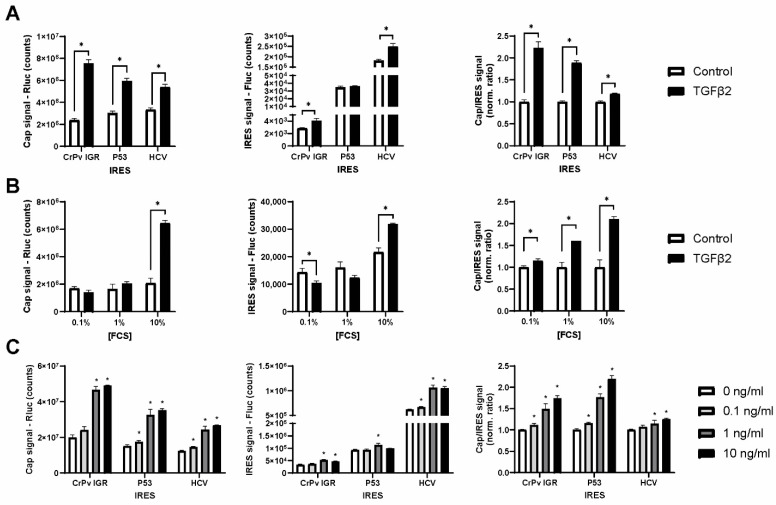
TGF-β2 specifically induces cap-mediated translation. SW1353 chondrocytic cells were treated for 3 days with 10 ng/mL TGF-β2 or medium control. In all experiments, cells were transfected with reporters at day 2 and measured 24 h later at day 3 post-stimulation. (**A**) The effect of TGF-β2 on activity of CrPv IGR, P53 and HCV IRES reporters. Cap-dependent Renilla luciferase (left panel), IRES-dependent firefly luciferase signal (middle panel) and cap/IRES ratio normalized to control (right panel). (**B**) Cells were stimulated with TGF-β2 in 0.1, 1, or 10% FCS. (**C**) Cells were stimulated with 0–10 ng/mL TGF-β2 in 10% FCS. Bar graphs show mean ± standard deviation (n = 3). Statistical analysis was performed with a *t*-test comparing the control to the TGF-β2 condition. * *p*-value < 0.05 when compared to control or 0 ng/mL.

Next, we tested whether TGF-β2 could affect ribosome modus by utilizing bicistronic reporter assays reporting on cap-mediated translation and activity of the CrpV IGR, P53 or HCV IRES elements. A specific stimulatory effect of TGF-β2 was found on cap-mediated translation (Figure 2A), while IRES-mediated translation was not (P53 IRES) or little affected (CrPv IGR, HCV IRES). The ratio of cap/IRES translation was significantly increased. Since TGF-β2 signaling is often studied in serum-starved cells, the requirement of FCS was evaluated for the effect on ribosome modus. Although the cap/IRES ratio was significantly increased under conditions of 0.1, 1 and 10% FCS, a convincing induction of the cap cistron was found only in the presence of 10% FCS (Figure 2B). These data suggest that TGF-β2 stimulation modifies existing protein translation activity in the presence of 10% FCS towards cap-mediated translation. Finally, the concentration of TGF-β2 was reduced to establish dose dependency of this effect. Both 1 ng/mL and 10 ng/mL increased the cap signal convincingly, while 0.1 ng/mL had only a marginal effect on the cap signal and cap/IRES ratio (Figure 2C). The lowest concentration of TGF-β2 was able to induce SMAD3 transcriptional activity but failed to induce target gene expression of SERPINE1 (Supplementary Figure S3A–C). This effect on cap-mediated translation was not identified at 1-day post-stimulation, but only following 3 days. Taking these together, we found that ≥1 ng/mL TGF-β2 is able to specifically enhance cap-mediated translation under standard culture conditions.

### 2.3. TGF-β2 Affects rRNA Processing and rRNA Post-Transcriptional Modifications

**Figure 3:**
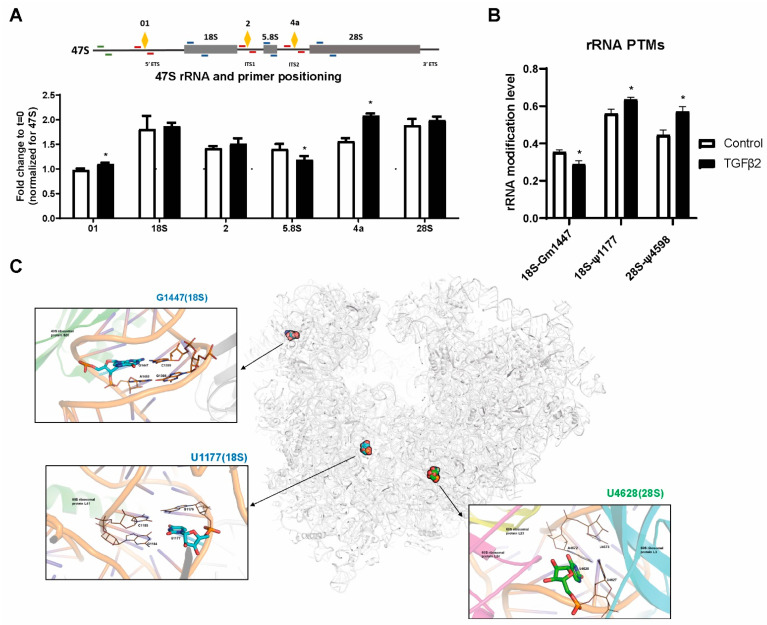
Ribosomal RNA 2′-O-methylation is decreased (18S-Gm1447) and pseudouridylation is increased (18S-ψ1177, 28S-ψ4598) by three days of TGF-β2 stimulation. (**A**) rRNA processing of the 47S primary rRNA transcript was probed by RT-qPCR. Primer pair positioning is indicated in the image of the 47S primary rRNA transcript. ETS = external transcribed spacer, ITS = internal transcribed spacer. Orange diamonds labelled 01 and 4a denote pre-rRNA processing cleavage sites, which are spanned by the primer pairs indicated in red. White bars: control treatment, black bars: TGF-β2 treament. Data were normalized to a full-length 47S rRNA precursor amplicon (green primer pair). * = *p*-value < 0.05. (**B**) RiboMethLevels and PsiScores of altered rRNA PTMs. All detected rRNA modifications can be found in Supplementary Figure S3. Modifications are mediated by SNORD127 [35] (18S-Gm1447) and SNORA17 [36] (28S-ψ4598). The guide snoRNA is not known for 18S-ψ1177. (**C**) Visualization of the human ribosome with the location of the three altered modification sites. Close-ups of the insets can be found in Supplementary Figure S5.

**Figure 4:**
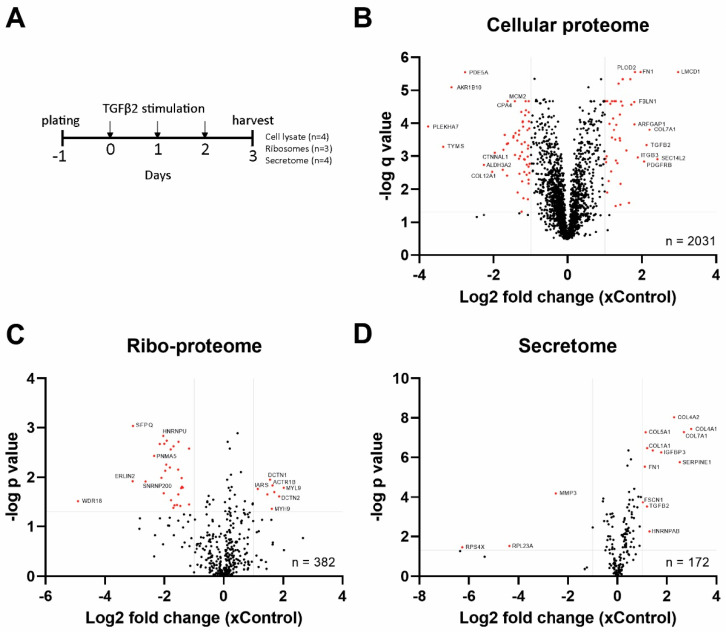
Multi-proteomic approach to identify differentially expressed proteins. (**A**) The experimental setup for cellular, secretome and ribosome proteomics. Volcano plots of all detected proteins in (**B**) isolated ribosome, (**C**) samples cell lysates, (**D**) medium samples. Each dot represents one detected protein. Proteins with FC ≥ 2.0 and *p*-value < 0.05 (ribosomal proteome and secretome) or *q*-value (cellular proteome) are depicted in red. Grey lines demarcate cut-off values.

Since we observed a specific effect on cap-mediated translation after 3 days, we analyzed the ribosome in more detail. Ribosome half-life has not been determined in articular cartilage, but in rat liver tissue, this was approximately 75 h [32]. Therefore, we tested whether TGF-β2 stimulation leads to changes in composition of the ribosomal pool. First, we probed rRNA expression and processing by RT-qPCR. The unprocessed 5′ external transcribed spacer (ETS) fragment (cleavage site 01) was slightly more abundant after TGF-β2 stimulation, while 5.8S rRNA decreased and internal transcribed spacer 2 (ITS2) fragment (cleavage site 4a) abundance increased (Figure 3A). Cleavage at site 4a and removal of the ITS2 from 5.8S pre-rRNA constitute one of the last steps of rRNA processing [33]. These data indicate that rRNA processing is altered by prolonged TGF-β2 stimulation. Ribosome heterogeneity can arise from variation in small nucleolar RNA (snoRNA)-mediated rRNA PTMs [34]. We determined rRNA 2′-O-methylation and ψ profiles in cells treated with or without TGF-β. We quantified 109 2′-O-me sites and 104 Ψ sites. 2′-O-me levels of 18S-G1447 decreased by TGF-β2 treatment, while 18S-Ψ1177 and 28S-Ψ4598 were significantly increased (Figure 4B). The positions of these rRNA PTMS were mapped to the human ribosome (Figure 3C, Supplementary Figure S5). In conclusion, very specific effects of TGF-β2 were found on rRNA expression, processing and modification.

### 2.4. TGF-β2 Alters Ribosome Protein Composition, Cellular Proteome and Secretome

**Figure 5:**
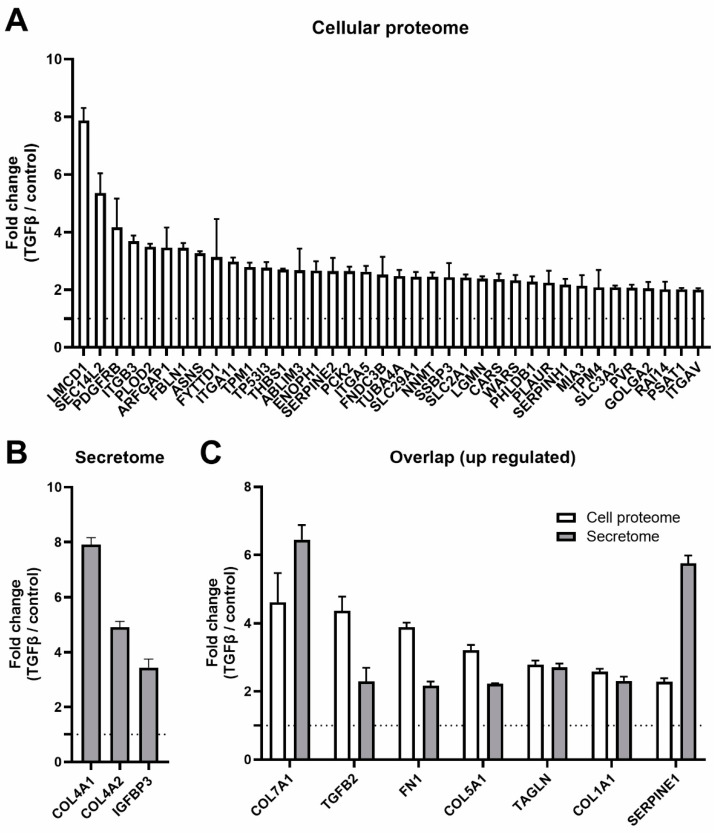
Upregulated proteins in the cellular proteome and secretome contain well-known TGF-β-target genes. (**A**) Proteins that were DE and upregulated by TGF-β2 in the cellular proteome. (**B**) Proteins that were DE and upregulated by TGF-β2 in the secretome. (**C**) Proteins that were DE and upregulated by TGF-β2 in the cellular proteome and the secretome. All proteins were significantly increased by TGF-β2 when compared to the control. Bar graphs show mean ± standard deviation (n = 4). Proteome analysis was done using one-way ANOVA, and FDR-adjusted *q*-values were used to assess statistical significance. Dashed lines denote the control expression level of 1.

Ribosome heterogeneity can also arise from variation in the protein composition of core ribosomal or accessory proteins. To assess the effects of TGF-β2 on ribosomal protein composition and its consequences for cellular protein profiles, an experiment was designed to analyze isolated ribosomes, cellular proteome and secreted proteins of SW1353 cells treated with TGF-β2 (Figure 4A). Low-salt washing of isolated ribosomes was used to retain ribosome-interacting proteins [37]. LC-MS/MS identified 382 proteins in the ribosomal proteome, of which 38 were differentially abundant in ribosomes of TGF-β2-treated cells (Fold change (FC) > 2.0 and *p*-value < 0.05) (Table 1; Figure 4B). Myosin light/heavy chain proteins (MYL9, MYH9) and Dynactin subunits 1 and 2 (DCTN1, DCTN2) were the most upregulated proteins in the ribosomal proteome. In cell lysates, 2031 proteins were identified and 115 were differentially expressed (DE) (FC > 2.0 and *q*-value < 0.05) (Table 1; Figure 4C). Of the 172 proteins identified in the secretome, 15 were DE in response to TGF-β2 treatment (FC > 2.0 and *p*-value < 0.05) (Table 1; Figure 4D). A strong increase of COL4A1, COL4A2 and IGFBP3 was detected in the TGF-β2 secretome (Figure 5B). We identified an overlap in proteins DE in cell lysate and the secretome of TGF-β2-stimulated cells, including well-known TGF-β target genes (e.g., *FN1*, *TGFB2*, *SERPINE1*, *COL1A1*, *COL5A1* and *COL7A1*) (Figure 5C). MYL9, identified as differentially abundant in TGF-β2-dependent ribosomal proteome, was also among the highest upregulated proteins in the cellular proteome (Figure 4B,C).

**Figure 6:**
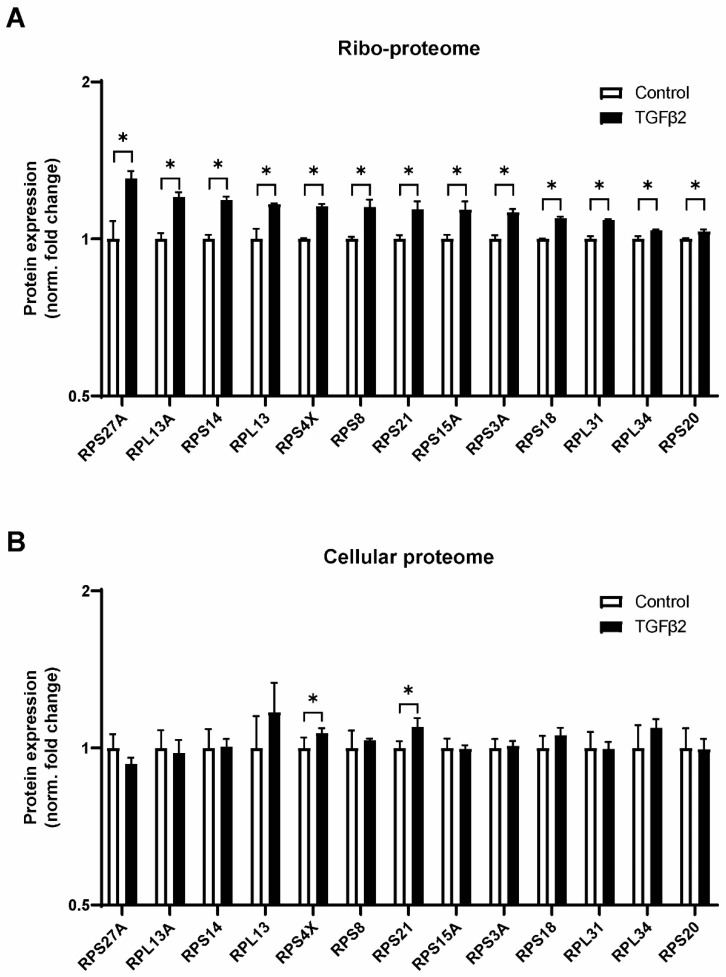
TGF-β2 alters ribosomal protein composition. (**A**) Proteome analysis of core ribosomal proteins after 3 days of control or TGF-β2 stimulation. (**B**) Expression changes in the cellular proteome are shown for the same proteins on the same scale for direct comparison. * = *q*-value < 0.05 in cellular proteome, *p*-value < 0.05 in ribosomal proteome.

Of the core ribosomal proteins, we identified 30 ribosomal proteins from the small subunit (RPSs) and 41 ribosome proteins from the large subunit (RPLs) in both cell lysates and isolated ribosomes (Figure 6A,B). The expression level of RPs between cell lysates and isolated ribosomes correlated well (r = 0.80, *p*-value < 0.0001) in both the control and TGF-β2 condition. Six RPs (RPL7L1, RPL8, FAU/RPS30, RPL36A, RPL36AL, RPL37) were identified only in the cell lysates and not in isolated ribosomes. In addition, RPLP1 was largely lost from isolated ribosomes when compared to cell lysates. RPLP1 plays a crucial role in translation elongation; thus, its absence in isolated ribosomes is likely caused by ribosome stalling due to cycloheximide treatment [38]. Cycloheximide was added to the cells prior to ribosome isolation to stall ribosomes and facilitate their isolation. Interestingly, we found that TGF-β2 treatment increased the abundance of nine RPSs (RPS3A, RPS4X, RPS8, RPS14, RPS15A, RPS18, RPS20, RPS21, RPS27A) and four RPLs (RPL13, RPL13A, RPL31, RPL34) in isolated ribosomes. Fold changes were relatively low and did not exceed 1.3-fold (Figure 6A). Nevertheless, these proteins were not differentially expressed in the cellular proteome, with the exception of RPS4X and RPS21 (Figure 6B), indicating their specific enrichment in TGF-β2-treated ribosome isolates. A total of 57 proteins were detected exclusively in the ribosomal proteome in comparison with the cellular proteome. Among these proteins were elongation factors (EEF1A1P5, EEF1E1), the eIF2B complex (EIF2B2, EIF2B3, EIF2B4 and EIF2B5), RPS27, proteins that are required for protein synthesis (DNAJC2 [39], DHX29 [40], DHX30 [41], FXR2 [42]), members of the ribosome quality control complex (LTN1, NEMF [43]) and the m6a (N6-methyladenosine) reader protein YTHCD2 [44]. These results confirm that the enrichment of ribosomes occurred and that the reduced complexity of the sample allows for LC-MS/MS identification of proteins that would otherwise remain undetectable.

**Figure 7:**
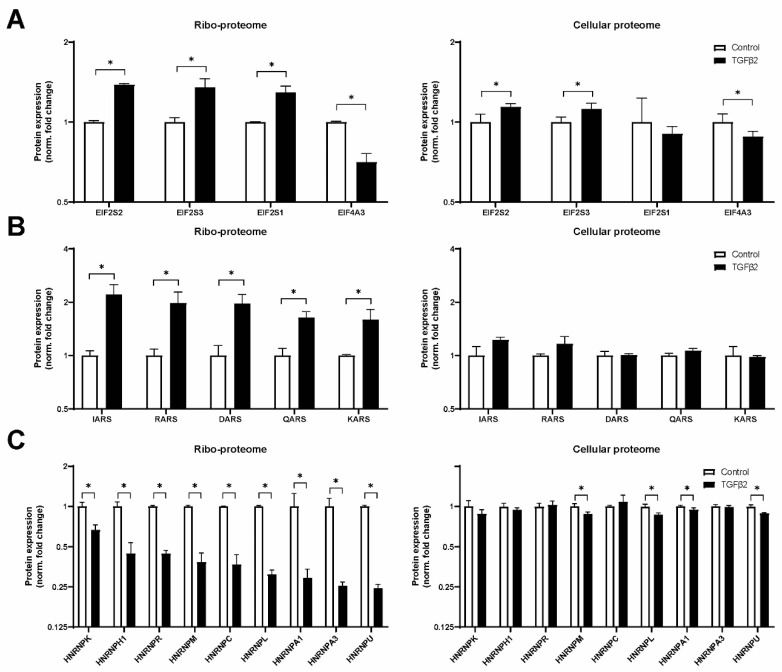
LC-MS/MS proteomics reveals enrichment of EIF2 and tRNA ligases and a concomitant reduction of HNRNPs in TGF-β2-stimulated ribosomes. Significantly regulated (**A**) eukaryotic initiation factor subunits, (**B**) tRNA ligases and (**C**) HNRNPs in isolated ribosomes (left panels). Expression changes in the cellular proteome are shown for the same proteins on the same scale to facilitate direct comparison (right panels). * = *p*-value < 0.05 in ribosomal proteome, *q*-value < 0.05 in cellular proteome.

Analyzing the ribosomal proteome, we also focused on initiation factors (Figure 7A). EIF2 is a crucial component of the pre-initiation complex that depends on EIF2B [45] and carries the initiator methionyl-transfer RNA (tRNA) to the translation start codon [46]. EIF2 subunits 1, 2 and 3 were 1.3–1.4-fold enriched in TGF-β2-stimulated ribosome samples. Although this is a modest fold change, it correlates well with the observed induction of total protein translation by TGF-β2 (cf. Figure 1A). EIF4A3 was 1.4-fold decreased in TGF-β2-stimulated cells. It is part of the exon junction complex that post-transcriptionally regulates translation [47]. In good agreement with increased amounts of eIF2, we found an increased level of tRNA ligases (1.6–2.2 fold) associated with ribosomes from TGF-β2-stimulated cells (Figure 7B). These five tRNA ligases catalyze the tRNA aminoacylation of the amino acids Ile, Arg, Asp, Gln and Lys. Finally, we filtered for IRES-transacting factors [18] in our dataset and found that 10 proteins from the Heterogeneous nuclear ribonucleoprotein (HNRNP) family were reduced 1.5–4.0-fold in the TGF-β2 riboproteome (Figure 7C). This observation is in agreement with a relative reduction in IRES-mediated translation (cf. Figure 2). Altogether, our data demonstrate that TGF-β2 stimulation induced changes in chondrocytes’ ribosome protein composition, with consequences for ribosome function/translation.

## 3. Discussion

In the present study, we aimed to elucidate the effects of TGF-β on ribosome activity, modus and composition. Prolonged TGF-β2 stimulation enhanced protein translation in SW1353 cells. A boost in protein synthesis was also observed in TGF-β2-treated HACs. The stimulatory effect on ribosome activity was specific to cap-mediated translation and required ≥1 ng/mL of TGF-β2. In addition, we found a modest effect on rRNA processing, especially 5.8S maturation and three differentially modified rRNA PTMsites: 18S-Gm1447, 18S-Ψ1177 and 28S-Ψ4598. Proteomic analyses of cellular proteome and secretome confirmed enhanced protein production and secretion of known TGF-β target gene products such as *SERPINE1*, *FN1*, *COL1A1*, *COL1A2* and *TGLN*. Ribosomal proteome analysis revealed an increased association of eIF2 and six tRNA ligases, and reduced interaction of HNRNPs with ribosomes of cells stimulated with TGF-β. These results matched well with the observed changes in TGF-β2-driven ribosome activity and modus.

Recently, it was shown that TGF-β1 induces protein synthesis of collagen type II and aggrecan through modulation of 4E-BP1 phosphorylation in HACs and explant cultures [6]. Our data are in agreement with the total protein translation data that were published by this group. However, it should be taken into account that we used the SW1353 cell line instead of HACs. While SW1353 is not a primary cell source, it did provide us the opportunity to work with larger cell numbers and provide sufficient material to isolate ribosomes and study their composition. Future work should further pinpoint our findings in primary human articular chondrocytes.

For rRNA biogenesis, we found a decreased abundance of 5.8S rRNA and increased levels of its 4a intermediate in the presence of TGF-β. The exact biological meaning of reduced 5.8S rRNA is not clear; however, this may indicate a less efficient maturation of 5.8S rRNA or a change in rRNA turnover [48]. Recent advances in RNA-sequencing technology have drastically improved methods to detect RNA nucleotide modifications, such as 2′-O-methylation and pseudouridylation [29,49]. The modification levels of 18S-Gm1447 were lower in 195 triple-negative breast cancer samples [50,51]. We found a decrease in 2′-O-me levels of this site in SW1353 cells after TGF-β2 stimulation. The snoRNA responsible for 18S-Gm1447 modification is SNORD127 [35]. 28S-Ψ4598 was increased by TGF-β2, and this modification is guided by SNORA17 [36]. Expression of this snoRNA was increased in 91 human hepatocellular carcinoma (HCC) samples [52], and TGF-β2 signaling is known to be involved in HCC [53]. Based on our results we thus expect a functional link between TGF-β2 and 28S-Ψ4598 in HCC or TGF-β2 and 18S-Gm1447 in breast cancer. The biological consequence of alterations in modification levels of many specific rRNA nucleotides remains to be determined.

Our multi-proteomics approach to investigate cellular proteomes, isolated ribosomes and secreted proteins provided an opportunity for data integration. The combination of cellular and secretome proteomics allowed the identification of additional TGF-β-target genes. For example, COL1A1, COL4A1 and COL5A1 were shown to be induced by TGF-β2 [54]. However, COL4A1 was not detected in the cellular proteome, yet reliably quantified in the secretome.

Mounting evidence indicates that variation in ribosomal protein composition is a significant contributor to ribosome heterogeneity and can affect ribosome function [21,23]. In our model, a 3-day TGF-β2 stimulation changed ribosome activity and modus; hence, it was opportune to evaluate ribosomal protein composition. We identified a very similar number of RPs (26 RPSs and 38 RPLs) in our cellular and ribosomal proteome as in a previous ribosome structure and mass spectrometry study [55]. We measured an increased association of nine RPSs and four RPLs with ribosomes of TGF-β2-stimulated cells. Notably, six of these proteins (RPS3A, RPS4X, RPS8, RPS14, RPS20 and RPL31) were upregulated in brain capillaries of Alzheimer’s disease patients [56]. Additional literature implicates *RPS3A* expression upregulation in Alzheimer’s disease [57] and poor prognosis in HCC [58]. RPSX4X overexpression was shown to boost cell growth and migration through mTOR/S6K1 [59]. Similar effects on cell growth were reported for RPS15A [60], RPS20 [61], RPS21 [62], RPS27A [63] and RPL34 [64]. RPS14 deficiency leads to anemia, while overexpression rescued erythropoiesis, and this was linked to TGF-β-mediated MMP-9 expression [65]. Mutations in RPL13 were found to cause severe skeletal dysplasia, and its expression was found to be localized to the hypertrophic zone in murine growth plate chondrocytes [66]. Taken together, RPs that we found to be increased in ribosome association following TGF-β2 stimulation have been associated with cell growth in various diseases, in particular cancers. Direct evidence for differences in translational properties of ribosomes of TGF-β2-treated cells might be obtained by utilization of a cell-free translation system [67]. Ribosome dynamics as a function of microenvironmental changes are little studied in mammalian cells. Nevertheless, a yeast study where 24 h nutrient deprivation was applied identified similar fold changes (~1.3) in the stoichiometry of eleven RPs by LC-MS/MS [68]. Future studies may elucidate whether small changes in RP stoichiometry in MS experiments can be validated at the structural level. In this study, we employed three well-studied IRES elements [69] and found that TGF-β2 stimulation alters the cap/IRES translation ratio in favor of the cap-mediated translation. This matched well with data obtained from ribosomal proteomics, where increased eIF2/tRNA ligases and reduced IRES trans-acting factors were found to be more associated with ribosomes following TGF-β2 stimulation. To our knowledge, such concordance between a bicistronic IRES reporter and ribosome proteomics has not been reported before.

The function of TGF-β2 in cartilage homeostasis and OA has been extensively studied [70]. However, its direct effect on protein synthesis was reported only recently [6]. Our data reveal that TGF-β2 modulate specific aspects of protein translation and ribosome composition in a chondrocytic cell type with potential consequences for preferential translation mechanisms. As such, the present work connects data on ribosome dysfunction in osteoarthritis [3] with TGF-β2. In future work, it would be relevant to determine whether TGF-β2 can normalize OA-related ribosome dysfunction in chondrocytes. This would position the ribosome as a druggable target for OA therapy, as is already the case for Alzheimer’s disease [71] and cancer [72].

## 4. Materials and Methods

### 4.1. Cell Isolation & Culture

SW1353 cells and primary human articular chondrocytes (HACs) were cultured in DMEM high glucose (Thermo Fisher Scientific, Breda, The Netherlands), supplemented with 10% fetal calf serum (FCS, Sigma-Aldrich, Zwijndrecht, The Netherlands) and 1% penicillin/streptomycin (P/S, Gibco, Breda, The Netherlands) at 37 °C in a humidified atmosphere with 5% CO_2_. Articular cartilage was resected from the subchondral bone and digested overnight in type II collagenase (300 U/mL) in DMEM/F12 HEPES and 1% P/S at 37 °C with continuous shaking. The digested cartilage solution was passed through a 70 μm cell strainer (Greiner Bio-One, Alphen aan de Rhijn, The Netherlands); cells were washed twice with 0.9% NaCl and plated at 30 k/cm^2^ in DMEM/F-12 (Thermo Fisher Scientific) containing 10% FCS, 1% P/S and 1% non-essential amino acids (Gibco) at 37 °C in a humidified atmosphere with 5% CO_2_. Upon confluency, HACs were passaged 1:2 until passage 2. Cells were stimulated with 10 ng/mL TGF-β2 (Gibco PHG9114). Similar results were obtained with TGF-β3 (Invitrogen RP8600) for total protein translation and ribosome modus.

### 4.2. Total Protein Translation Measurements

SW1353 cells were incubated in 6-well plates for 30 min with 25 µCi/mL EasyTag [35S] protein labeling mix (Perkin Elmer, Rotterdam, The Netherlands) in DMEM without methionine or cystine, and supplemented with 10% FCS and 1% P/S. Cells were carefully washed four times with 0.9% NaCl and lysed in 150 µL RIPA buffer. Lysates were sonicated in a water bath and centrifuged for 15 min at 13,200 rpm in an Eppendorf tabletop centrifuge. A quantity of 50 µL of cleared lysates was measured for 5 min on a scintillation counter (Tri-Carb 2910 TR, Perkin Elmer, Rotterdam, The Netherlands), and data were extracted as counts per minute beta. Data were normalized to total DNA content per well using a Sybr Green DNA assay as described earlier [73]. Alternatively, SW1353 cells were incubated in a 96-well plate for 24 h with 50 µM of the methionine analogue homopropargylglycine (HPG) in DMEM without methionine and cystine, and supplemented with 63 mg/L cystine (Sigma-Aldrich), 10% FCS and 1% P/S. HPG incorporation was detected with a Click-IT reaction (HPG Alexa Fluor™ 488 Protein Synthesis Assay Kit, Thermo Fisher Scientific, Breda, The Netherlands) with a TriStar2 (Berthold Technologies, Vilvoorde, Belgium). Data were normalized to total DNA content per well using the included HCS Nuclear mask blue stain.

### 4.3. Cell Transfection and Luciferase Reporter Assays

Media in 24-well plates were replaced by 0.45 mL normal culture medium without P/S per well. Next, a transfection mix was made containing 50 µL HEPES buffered DMEM/F12, 0.5 µg plasmid DNA and 1.5 µL Fugene6 (Promega, E2693, Leiden, The Netherlands) per well and complexed for 20 min. Transfected plasmid DNA consisted of 90% IRES or CAGA12 reporter and 10% pCDNA4-LACZ to correct for transfection efficiency. The Rluc/Fluc IRES constructs containing the CrPV IGR, P53 and HCV IRES were a kind gift from Dr. Sunnie Thompson (see Supplementary Figures S7–S9 for construct details). The CAGA12 reporter construct was a gift from Dr. Tamas Dalmay. Subsequently, the transfection mix was added drop-wise to the wells, incubated for 4 h, and media were then supplemented with 0.5 ml of 2% P/S containing normal culture medium with or without 20 ng/mL TGF-β2 and incubated for an additional 20 h. Cells were washed once with 0.9% NaCl, lysed in 100 µL passive lysis buffer per well for 15 min at room temperature on a flat orbital shaker (IKA HS260, Sigma-Aldrich, Zwijndrecht, The Netherlands). Lysates were detached by pipetting up and down three times and transferred to 0.5 mL Eppendorf tubes. Tubes were centrifuged for 10 min at 16,550× *g* in a tabletop Eppendorf centrifuge. Fifty µL of lysate was added to black fluotrac plates and used for dual luciferase measurements using Promega’s dual luciferase assay system. Substrates were injected (50 µL/well) and the signal was detected with a Tristar2 injection system (Berthold Technologies, Vilvoorde, Belgium). Twenty µL lysate was used to detect β-galactosidase activity using a β-Gal assay kit (Invitrogen, Breda, The Netherlands).

### 4.4. RT-qPCR and rRNA PTM Quantification

Total RNA was isolated with the RNeasy kit (Qiagen, Venlo, The Netherlands) according to the manufacturer’s instructions. Reverse transcription and gene expression analyses were performed as described in detail elsewhere [74]. Primer pairs used for RT-qPCR are shown in Table 2. Data were normalized to a full-length 47S rRNA precursor for ribosomal RNA (rRNA) and rRNA intermediates, while for regular gene expression, data were normalized to *Peptidylprolyl Isomerase A* (*PPIA*). Ribosomal RNA post-transcriptional modifications (PTMs) were determined by RiboMethSeq and HydraPsiSeq as described earlier [29,75].

### 4.5. Ribosome Isolation

Cytoplasmic fractions were generated as described earlier [76]. SW1353 cells were incubated with 100 µg/mL cycloheximide (CHX, Sigma-Aldrich, Zwijndrecht, The Netherlands) for 5 min at 37 °C, washed twice with cold 0.9% NaCl containing 100 µg/mL CHX and harvested with a rubber policeman. Cells were centrifuged for 5 min at 284× *g* in a Hettich Rotanta 460S centrifuge and resuspended in 4× pellet volume of Cytoplasmic extraction buffer (20 mM Tris pH 7.5, 5 mM MgCl_2_, 10 mM NaCl, 0.15% NP-40) with fresh addition of Roche protease inhibitor cocktail (1×), 100 µg/mL CHX and 40 U/mL RNasin (Promega, Leiden, The Netherlands). Cell membranes were disrupted by 40 full strokes with a Dounce homogenizer per sample, and membrane rupture was verified by Trypan blue staining of a small volume of the sample. Nuclei were pelleted by centrifugation at 1370× *g* for 10 min in a tabletop centrifuge at 4 °C, and the supernatant was transferred to new pre-cooled Eppendorf tubes. This supernatant was centrifuged at 16,550× *g* for 10 min at 4 °C to pellet mitochondria. The resulting post-mitochondrial (PMT) fraction was measured on a Nanodrop, and 125 µg RNA was loaded onto a sucrose cushion [77]. Successful fractionation of cells was determined by immunoblotting (Supplementary Figure S6A). The sucrose cushion consisted of 4 mL 1 M sucrose, 4 mL 0.7 M sucrose and 3 mL cytoplasmic extraction buffer in Seton open-top polyclear ultracentrifuge tubes (part nr 7030). Sucrose solutions were prepared in 20 mM Tris pH 7.5, 5 mM MgCl_2_ and 25 mM NaCl from a 2.2 M sucrose stock solution, passed through 0.22 µm filters (Millipore, Amsterdam, The Netherlands) and degassed with a vacuum before use. Samples were centrifuged at 120,000× *g* at 4 °C for 17 h in an SW41Ti rotor (Beckman, Woerden, The Netherlands) with maximum acceleration and brake setting 9. Pellets were resuspended in either RLT buffer for RNA isolation, RIPA buffer with 1× Roche protease inhibitors for immunoblotting or 25 mM ammonium bicarbonate (Sigma-Aldrich) in LC-MS grade water (Pierce, Amsterdam, The Netherlands) with 1× Roche protease inhibitors for liquid chromatography tandem mass spectrometry (LC-MS/MS). Successful isolation of ribosomes was validated by immunoblotting and total RNA agarose gel electrophoresis (Supplementary Figure S6A,B).

### 4.6. Immunoblotting

Nuclear and mitochondrial pellets were resuspended in an appropriate volume of 25 mM ammonium bicarbonate. All fractions were mixed 1:1 with RIPA lysis buffer (50 mM Tris-HCl pH 8.0, 150 mM NaCl, 1% NP-40, 0.5% DOC, 0.1% SDS, 5 mM EDTA) containing 2× Roche protease inhibitor cocktail. Samples were sonicated with 12 cycles, 1 s on, 1 s off using a Soniprep 150 rod sonicator at amplitude 6. Next, samples were centrifuged for 10 min at 16,550× *g* at 4 °C, and the supernatant was transferred to new Eppendorf tubes. An equal amount of lysate was boiled with sample buffer for 5 min at 95 °C and separated on a 4–20% precast gradient gel (Bio-Rad, Tokyo, Japan). For p-mTOR and p-4E-BP1, an equal amount of protein (40 µg) was loaded per lane. Samples were transferred to nitrocellulose membranes, blocked in TBS with 0.1% NP-40 and 5% (*w*/*v*) non-fatty dry milk powder. Primary antibodies were anti-phospho-mTOR Ser2448 (Cell signaling technologies, D9C2, Danvers, MA, USA), anti-phospho-4E-BP1 Thr37/46 (Cell signaling technologies, 236B4), anti-GAPDH (Fitzgerald, 4G5, Compton, UK), anti-α-Tubulin clone (Sigma, B512), anti-RPL7 (Novus bio, NB100-2269, Littleton, CO, USA) or anti-Histone H3 (Abcam, 24834, Cambridge, UK). Secondary antibodies were rabbit anti-mouse ALP or goat anti-rabbit ALP (both from DAKO, Glostrup Kommune, Denmark), and these were detected with CDP-STAR (Applied Biosystem, Breda, The Netherlands) on a Bio-Rad Chemidoc MP.

### 4.7. Agarose Gel Electrophoresis of Total RNA

A quantity of 7 µL from each RNA isolate was mixed with loading buffer (10× stock consisting of 50% glycerol and 0.5% Orange G) and run for 1 h at 100 V on a 1% Agarose TBE gel with ethidium bromide (0.4 mg/L). The most intense nuclear sample corresponded to 7.5 µg of total RNA. Images were acquired with a Bio-Rad Chemidoc MP.

### 4.8. LC-MS/MS Proteomics

Sample quality was assessed by running 7.5 µg protein on a NuPAGE 4–12% Bis-Tris protein gel followed by Coomassie blue staining (Supplementary Figure S6C). A quantity of 100 µg of each secretome sample was taken into low-binding tubes (LoBind, Thermo Fisher Scientific, Breda, The Netherlands). Volume was brought up to 1 mL using LC-MS water (Pierce), and 10 µL of Strataclean resin was added to each sample. Samples were vortexed for 1 min and centrifuged for 1 min at 2000 rpm. The supernatant was removed, and the pellet was washed with 1 mL of LC-MS water, vortexed and centrifuged. The supernatant was removed, and the wash step was repeated. Finally, all supernatant was carefully removed, and the pellet was resuspended in 160 μL of 25 mM ammonium bicarbonate. One hundred µg of proteome and riboproteome sample was taken, and the final volume was brought up to 160 µL using 25 mM Ambic. The proteome, riboproteome and secretome samples were prepared at the same time. Within each group, samples were randomized for sample preparation. A quantity of 10 µL of 1% (*w*/*v*) Rapigest was added and heated at 80 °C for 10 min. A quantity of 10 μL of 11 mg/mL solution of DTT (4 mM final) was added and incubated at 60 °C for 10 min. A quantity of 10 µL of 46.6 mg/mL solution of iodoacetamide (14 mM final) was added and incubated at room temperature for 30 min. Any excess iodoacetamide was quenched by adding 9.4 µL of DTT used in the previous step. A quantity of 10 µL of 0.2 µg/µL trypsin/lys-c was added and samples were incubated overnight at 37 °C in the rotating incubator. On the next day, the same amount of trypsin/lyc-C was added, and samples were incubated for 2 more hours at the same conditions. Secretome samples were centrifuged at 17,000× *g* for 15 min, and the supernatant was transferred to a new LoBind tube. To all samples, 1 µL of trifluoroacetic acid was added, and samples were then incubated for 45 min at 37 °C. Samples were centrifuged for 15 min at 17,000× *g* at 4 °C, and the supernatant was transferred to a new LoBind tube (secretome samples were centrifuged twice). A quantity of 500 ng of each tryptic digest was subjected to LC-MS/MS, using a 2 h gradient for cellular proteomics, 1 h gradient for secretome proteomics and half an hour gradient for the ribosomal proteomics. Data-dependent analyses were conducted on a QExactive HF quadrupole-Orbitrap mass spectrometer coupled to a Dionex Ultimate 3000 RSLC nano-liquid chromatograph (Hemel Hempstead, UK). Loading of the column and mass spectrometer data acquisition were done as described earlier [78]. Progenesis QI software (Waters, Manchester, UK) and our local Mascot server (Version 2.6.2) were used for label-free quantification against the Unihuman Reviewed database with carbamidomethyl cysteine as a fixed modification and methionine oxidation as a variable modification, peptide mass tolerance of 10 ppm and fragment tolerance of 0.01 Da. Proteins identified with only one unique peptide were discarded a priori from further analyses.

### 4.9. Statistics

Two group comparisons were done with two-tailed Student’s *t*-tests (Figure 1, Figure 2 and Figure 3). A normal distribution was assumed. For MS data, we utilized one-way ANOVA, FDR-adjusted q-values for the cellular proteome or *p*-values for the ribosomal proteome and cellular secretome. The latter was done due to the low number of detected proteins in those datasets (Table 2).

## Figures and Tables

**Table 1:** Summary of proteomic analyses of control and TGF-β2-treated cells. # = number, ↑ = up-regulated, ↓ = down-regulated.

Dataset	# Proteins Identified	Significantly Changed (TGF-β2/Control)	Fold Change > 2.0 (TGF-β2/Control)
Cellular proteome	2031	993 (387↑, 606↓)	115 (52↑, 63↓)
Ribosomal proteome	382	66 (30↑, 36↓)	38 (8↑, 30↓)
Secretome	172	89 (63↑, 26↓)	15 (12↑, 3↓)

**Table 2:** RT-qPCR primer pairs.

Primer	Forward Primer (5′-3′)	Reverse Primer (5′-3′)	Slope
47S	GTCGGAGAGGTTGGGCCT	GAGTCGGGACGCTCGGA	−3.37
47S 01	TGTCAGGCGTTCTCGTCTC	AGAGCACGACGTCACCACA	−3.58
18S rRNA	CGGACCAGAGCGAAAGCA	ACCTCCGACTTTCGTTCTTGATT	−3.42
47S 02	TGTGAAACCTTCCGACCCCTCT	CGAGTGATCCACCGCTAAGAGTCGTA	−3.70
5.8S rRNA	CACTCGGCTCGTGCGTCGAT	CGCTCAGACAGGCGTAGCCC	−3.80
47S 4a	CTAAGCGCAGACCCGGC	GTCTCTCTCAGCCGGGC	−3.00
28S rRNA	GCCATGGTAATCCTGCTCAGTAC	GCTCCTCAGCCAAGCACATAC	−3.50
SERPINE1	GTCTGCTGTGCACCATCCCCCATC	TTGTCATCAATCTTGAATCCCATA	−3.67
PPIA	TTCCTCCTTTCACAGAATTATTCCA	CCGCCAGTGCCATTATGG	−3.42

## Data Availability

Secretome mass-spec data was deposited: Project Name: Secretome of SW1353 cells cultured in the presence of TGF-β3, Project accession: PXD046109, Project DOI: 10.6019/PXD046109. Cellular proteome mass-spec data was deposited: Project Name: Cellular proteome of SW1353 cells cultured in the presence of TGF-β3, Project accession: PXD046111, Project DOI: 10.6019/PXD046111. Ribosomal proteome mass-spec data was deposited: Project Name: Ribosomal proteome of SW1353 cells cultured in the presence of TGF-β3, Project accession: PXD046114, Project DOI: 10.6019/PXD046114.

## Supplementary Materials

The following supporting information can be downloaded at: https://www.mdpi.com/article/10.3390/ijms25095031/s1.

## References

[1] Van der Kraan P.M., Berenbaum F., Blanco F.J., de Bari C., Lafeber F., Hauge E., Higginbottom A., Ioan-Facsinay A., Loughlin J., Meulenbelt I. (2016). Translation of clinical problems in osteoarthritis into pathophysiological research goals. RMD Open.

[2] Wallace I.J., Worthington S., Felson D.T., Jurmain R.D., Wren K.T., Maijanen H., Woods R.J., Lieberman D.E. (2017). Knee osteoarthritis has doubled in prevalence since the mid-20th century. Proc. Natl. Acad. Sci. USA.

[3] Van den Akker G.G.H., Caron M.M.J., Peffers M.J., Welting T.J.M. (2022). Ribosome dysfunction in osteoarthritis. Curr. Opin. Rheumatol..

[4] Shore D., Albert B. (2022). Ribosome biogenesis and the cellular energy economy. Curr. Biol..

[5] Katsara O., Kolupaeva V. (2018). mTOR-mediated inactivation of 4E-BP1, an inhibitor of translation, precedes cartilage degeneration in rat osteoarthritic knees. J. Orthop. Res. Off. Publ. Orthop. Res. Soc..

[6] Hwang H.S., Lee M.H., Kim H.A. (2020). TGF-β1-induced expression of collagen type II and ACAN is regulated by 4E-BP1, a repressor of translation. FASEB J..

[7] Caron M.M.J., Eveque M., Cillero-Pastor B., Heeren R.M.A., Housmans B., Derks K., Cremers A., Peffers M.J., van Rhijn L.W., van den Akker G. (2021). Sox9 Determines Translational Capacity During Early Chondrogenic Differentiation of ATDC5 Cells by Regulating Expression of Ribosome Biogenesis Factors and Ribosomal Proteins. Front. Cell Dev. Biol..

[8] Steinbusch M.M.F., van den Akker G.G.H., Cremers A., Witlox A.M.A., Staal H.M., Peffers M.J., van Rhijn L.W., Caron M.M.J., Welting T.J.M. (2022). Adaptation of the protein translational apparatus during ATDC5 chondrogenic differentiation. Non-Coding RNA Res..

[9] Wang W., Chun H., Baek J., Sadik J.E., Shirazyan A., Razavi P., Lopez N., Lyons K.M. (2019). The TGFβ type I receptor TGFβRI functions as an inhibitor of BMP signaling in cartilage. Proc. Natl. Acad. Sci. USA.

[10] Shao F., Liu X., Zhang X., Wang Q., Wang W. (2021). Methylation of 45S Ribosomal DNA (rDNA) Is Associated with Cancer and Aging in Humans. Int. J. Genom..

[11] MacInnes A.W. (2016). The role of the ribosome in the regulation of longevity and lifespan extension. WIREs RNA.

[12] Lomas C., Tang X.D., Chanalaris A., Saklatvala J., Vincent T.L. (2011). Cyclic mechanical load causes global translational arrest in articular chondrocytes: A process which is partially dependent upon PKR phosphorylation. Eur. Cells Mater..

[13] Burleigh A., Chanalaris A., Gardiner M.D., Driscoll C., Boruc O., Saklatvala J., Vincent T.L. (2012). Joint immobilization prevents murine osteoarthritis and reveals the highly mechanosensitive nature of protease expression in vivo. Arthritis Rheum..

[14] Madej W., van Caam A., Blaney Davidson E., Buma P., van der Kraan P.M. (2016). Unloading results in rapid loss of TGFβ signaling in articular cartilage: Role of loading-induced TGFβ signaling in maintenance of articular chondrocyte phenotype?. Osteoarthr. Cartil..

[15] Madej W., van Caam A., Davidson E.N., Hannink G., Buma P., van der Kraan P.M. (2016). Ageing is associated with reduction of mechanically-induced activation of Smad2/3P signaling in articular cartilage. Osteoarthr. Cartil..

[16] Watanabe M., Campbell T.M., Reilly K., Uhthoff H.K., Laneuville O., Trudel G. (2021). Bone replaces unloaded articular cartilage during knee immobilization. A longitudinal study in the rat. Bone.

[17] Yamamoto H., Unbehaun A., Spahn C.M.T. (2017). Ribosomal Chamber Music: Toward an Understanding of IRES Mechanisms. Trends Biochem. Sci..

[18] Godet A.C., David F., Hantelys F., Tatin F., Lacazette E., Garmy-Susini B., Prats A.C. (2019). IRES Trans-Acting Factors, Key Actors of the Stress Response. Int. J. Mol. Sci..

[19] Woodell-May J.E., Sommerfeld S.D. (2020). Role of Inflammation and the Immune System in the Progression of Osteoarthritis. J. Orthop. Res. Off. Publ. Orthop. Res. Soc..

[20] Del Carlo M., Loeser R.F. (2008). Cell death in osteoarthritis. Curr. Rheumatol. Rep..

[21] Gay D.M., Lund A.H., Jansson M.D. (2022). Translational control through ribosome heterogeneity and functional specialization. Trends Biochem. Sci..

[22] Genuth N.R., Barna M. (2018). Heterogeneity and specialized functions of translation machinery: From genes to organisms. Nat. Rev. Genet..

[23] Norris K., Hopes T., Aspden J.L. (2021). Ribosome heterogeneity and specialization in development. Wiley Interdiscip. Rev. RNA.

[24] Sonneveld S., Verhagen B.M.P., Tanenbaum M.E. (2020). Heterogeneity in mRNA Translation. Trends Cell Biol..

[25] Genuth N.R., Barna M. (2018). The Discovery of Ribosome Heterogeneity and Its Implications for Gene Regulation and Organismal Life. Mol. Cell.

[26] Jansson M.D., Häfner S.J., Altinel K., Tehler D., Krogh N., Jakobsen E., Andersen J.V., Andersen K.L., Schoof E.M., Ménard P. (2021). Regulation of translation by site-specific ribosomal RNA methylation. Nat. Struct. Mol. Biol..

[27] Genuth N.R., Shi Z., Kunimoto K., Hung V., Xu A.F., Kerr C.H., Tiu G.C., Oses-Prieto J.A., Salomon-Shulman R.E.A., Axelrod J.D. (2022). A stem cell roadmap of ribosome heterogeneity reveals a function for RPL10A in mesoderm production. Nat. Commun..

[28] Henras A.K., Plisson-Chastang C., Humbert O., Romeo Y., Henry Y. (2017). Synthesis, Function, and Heterogeneity of snoRNA-Guided Posttranscriptional Nucleoside Modifications in Eukaryotic Ribosomal RNAs. Enzymes.

[29] Marchand V., Pichot F., Neybecker P., Ayadi L., Bourguignon-Igel V., Wacheul L., Lafontaine D.L.J., Pinzano A., Helm M., Motorin Y. (2020). HydraPsiSeq: A method for systematic and quantitative mapping of pseudouridines in RNA. Nucleic Acids Res..

[30] Georgeson J., Schwartz S. (2021). The ribosome epitranscriptome: Inert-or a platform for functional plasticity?. RNA.

[31] Heldin C.H., Moustakas A. (2016). Signaling Receptors for TGF-β Family Members. Cold Spring Harb. Perspect. Biol..

[32] Nikolov E.N., Dineva B.B., Dabeva M.D., Nikolov T.K. (1987). Turnover of ribosomal proteins in regenerating rat liver after partial hepatectomy. Int. J. Biochem..

[33] Aubert M., O’Donohue M.F., Lebaron S., Gleizes P.E. (2018). Pre-Ribosomal RNA Processing in Human Cells: From Mechanisms to Congenital Diseases. Biomolecules.

[34] Motorin Y., Quinternet M., Rhalloussi W., Marchand V. (2021). Constitutive and variable 2′-O-methylation (Nm) in human ribosomal RNA. RNA Biol..

[35] Yang J.H., Zhang X.C., Huang Z.P., Zhou H., Huang M.B., Zhang S., Chen Y.Q., Qu L.H. (2006). snoSeeker: An advanced computational package for screening of guide and orphan snoRNA genes in the human genome. Nucleic Acids Res..

[36] Kiss A.M., Jády B.E., Bertrand E., Kiss T. (2004). Human box H/ACA pseudouridylation guide RNA machinery. Mol. Cell. Biol..

[37] Belin S., Hacot S., Daudignon L., Therizols G., Pourpe S., Mertani H.C., Rosa-Calatrava M., Diaz J.-J. (2010). Purification of Ribosomes from Human Cell Lines. Curr. Protoc. Cell Biol..

[38] Baliga B.S., Pronczuk A.W., Munro H.N. (1969). Mechanism of Cycloheximide Inhibition of Protein Synthesis in a Cell-free System Prepared from Rat Liver. J. Biol. Chem..

[39] Otto H., Conz C., Maier P., Wölfle T., Suzuki C.K., Jenö P., Rücknagel P., Stahl J., Rospert S. (2005). The chaperones MPP11 and Hsp70L1 form the mammalian ribosome-associated complex. Proc. Natl. Acad. Sci. USA.

[40] Sweeney T.R., Dhote V., Guca E., Hellen C.U.T., Hashem Y., Pestova T.V. (2021). Functional role and ribosomal position of the unique N-terminal region of DHX29, a factor required for initiation on structured mammalian mRNAs. Nucleic Acids Res..

[41] Pisareva V.P., Pisarev A.V. (2016). DHX29 and eIF3 cooperate in ribosomal scanning on structured mRNAs during translation initiation. RNA.

[42] Laggerbauer B., Ostareck D., Keidel E.M., Ostareck-Lederer A., Fischer U. (2001). Evidence that fragile X mental retardation protein is a negative regulator of translation. Hum. Mol. Genet..

[43] Tseng Y.J., Krans A., Malik I., Deng X., Yildirim E., Ovunc S., Tank E.M.H., Jansen-West K., Kaufhold R., Gomez N.B. (2024). Ribosomal quality control factors inhibit repeat-associated non-AUG translation from GC-rich repeats. Nucleic Acids Res..

[44] Kretschmer J., Rao H., Hackert P., Sloan K.E., Höbartner C., Bohnsack M.T. (2018). The m(6)A reader protein YTHDC2 interacts with the small ribosomal subunit and the 5′-3′ exoribonuclease XRN1. RNA.

[45] Vanselow S., Neumann-Arnold L., Wojciech-Moock F., Seufert W. (2022). Stepwise assembly of the eukaryotic translation initiation factor 2 complex. J. Biol. Chem..

[46] Adomavicius T., Guaita M., Zhou Y., Jennings M.D., Latif Z., Roseman A.M., Pavitt G.D. (2019). The structural basis of translational control by eIF2 phosphorylation. Nat. Commun..

[47] Hir H.L., Saulière J., Wang Z. (2016). The exon junction complex as a node of post-transcriptional networks. Nat. Rev. Mol. Cell Biol..

[48] Gingras A.C., Gygi S.P., Raught B., Polakiewicz R.D., Abraham R.T., Hoekstra M.F., Aebersold R., Sonenberg N. (1999). Regulation of 4E-BP1 phosphorylation: A novel two-step mechanism. Genes Dev..

[49] Marchand V., Blanloeil-Oillo F., Helm M., Motorin Y. (2016). Illumina-based RiboMethSeq approach for mapping of 2′-O-Me residues in RNA. Nucleic Acids Res..

[50] Kumari K., Groza P., Aguilo F. (2021). Regulatory roles of RNA modifications in breast cancer. NAR Cancer.

[51] Marcel V., Kielbassa J., Marchand V., Natchiar K.S., Paraqindes H., Nguyen Van Long F., Ayadi L., Bourguignon-Igel V., Lo Monaco P., Monchiet D. (2020). Ribosomal RNA 2′O-methylation as a novel layer of inter-tumour heterogeneity in breast cancer. NAR Cancer.

[52] McMahon M., Contreras A., Holm M., Uechi T., Forester C.M., Pang X., Jackson C., Calvert M.E., Chen B., Quigley D.A. (2019). A single H/ACA small nucleolar RNA mediates tumor suppression downstream of oncogenic RAS. eLife.

[53] Wang H., Wang P., Xu M., Song X., Wu H., Evert M., Calvisi D.F., Zeng Y., Chen X. (2021). Distinct functions of transforming growth factor-β signaling in c-MYC driven hepatocellular carcinoma initiation and progression. Cell Death Dis..

[54] Tatomir A., Tegla C.A., Martin A., Boodhoo D., Nguyen V., Sugarman A.J., Mekala A., Anselmo F., Talpos-Caia A., Cudrici C. (2018). RGC-32 regulates reactive astrocytosis and extracellular matrix deposition in experimental autoimmune encephalomyelitis. Immunol. Res..

[55] Van de Waterbeemd M., Tamara S., Fort K.L., Damoc E., Franc V., Bieri P., Itten M., Makarov A., Ban N., Heck A.J.R. (2018). Dissecting ribosomal particles throughout the kingdoms of life using advanced hybrid mass spectrometry methods. Nat. Commun..

[56] Suzuki M., Tezuka K., Handa T., Sato R., Takeuchi H., Takao M., Tano M., Uchida Y. (2022). Upregulation of ribosome complexes at the blood-brain barrier in Alzheimer’s disease patients. J. Cereb. Blood Flow Metab. Off. J. Int. Soc. Cereb. Blood Flow Metab..

[57] Tao Y., Han Y., Yu L., Wang Q., Leng S.X., Zhang H. (2020). The Predicted Key Molecules, Functions, and Pathways That Bridge Mild Cognitive Impairment (MCI) and Alzheimer’s Disease (AD). Front. Neurol..

[58] Zhou C., Weng J., Liu C., Zhou Q., Chen W., Hsu J.L., Sun J., Atyah M., Xu Y., Shi Y. (2020). High RPS3A expression correlates with low tumor immune cell infiltration and unfavorable prognosis in hepatocellular carcinoma patients. Am. J. Cancer Res..

[59] Bi G., Zhu D., Bian Y., Huang Y., Zhan C., Yang Y., Wang Q. (2021). Knockdown of GTF2E2 inhibits the growth and progression of lung adenocarcinoma via RPS4X in vitro and in vivo. Cancer Cell Int..

[60] Liu T., Zhang J., Chen H., Bianba T., Pan Y., Wang X., Jiang Y., Yang Z. (2022). PSMC2 promotes the progression of gastric cancer via induction of RPS15A/mTOR pathway. Oncogenesis.

[61] Krishnan R., Boddapati N., Mahalingam S. (2018). Interplay between human nucleolar GNL1 and RPS20 is critical to modulate cell proliferation. Sci. Rep..

[62] Wang T., Wang Z.Y., Zeng L.Y., Gao Y.Z., Yan Y.X., Zhang Q. (2020). Down-Regulation of Ribosomal Protein RPS21 Inhibits Invasive Behavior of Osteosarcoma Cells Through the Inactivation of MAPK Pathway. Cancer Manag. Res..

[63] Li H., Zhang H., Huang G., Bing Z., Xu D., Liu J., Luo H., An X. (2022). Loss of RPS27a expression regulates the cell cycle, apoptosis, and proliferation via the RPL11-MDM2-p53 pathway in lung adenocarcinoma cells. J. Exp. Clin. Cancer Res. CR.

[64] Ji P., Wang L., Liu J., Mao P., Li R., Jiang H., Lou M., Xu M., Yu X. (2019). Knockdown of RPL34 inhibits the proliferation and migration of glioma cells through the inactivation of JAK/STAT3 signaling pathway. J. Cell. Biochem..

[65] Youn M., Huang H., Chen C., Kam S., Wilkes M.C., Chae H.D., Sridhar K.J., Greenberg P.L., Glader B., Narla A. (2019). MMP9 inhibition increases erythropoiesis in RPS14-deficient del(5q) MDS models through suppression of TGF-β pathways. Blood Adv..

[66] Le Caignec C., Ory B., Lamoureux F., O’Donohue M.F., Orgebin E., Lindenbaum P., Téletchéa S., Saby M., Hurst A., Nelson K. (2019). RPL13 Variants Cause Spondyloepimetaphyseal Dysplasia with Severe Short Stature. Am. J. Hum. Genet..

[67] Penzo M., Carnicelli D., Montanaro L., Brigotti M. (2016). A reconstituted cell-free assay for the evaluation of the intrinsic activity of purified human ribosomes. Nat. Protoc..

[68] Samir P., Browne C.M., Rahul, Sun M., Shen B., Li W., Frank J., Link A.J. (2018). Identification of Changing Ribosome Protein Compositions using Mass Spectrometry. Proteomics.

[69] Weisser M., Schäfer T., Leibundgut M., Böhringer D., Aylett C.H.S., Ban N. (2017). Structural and Functional Insights into Human Re-initiation Complexes. Mol. Cell.

[70] Van der Kraan P.M. (2017). The changing role of TGFβ in healthy, ageing and osteoarthritic joints. Nat. Rev. Rheumatol..

[71] Rapaka D., Bitra V.R., Challa S.R., Adiukwu P.C. (2022). mTOR signaling as a molecular target for the alleviation of Alzheimer’s disease pathogenesis. Neurochem. Int..

[72] Pellegrino S., Meyer M., Zorbas C., Bouchta S.A., Saraf K., Pelly S.C., Yusupova G., Evidente A., Mathieu V., Kornienko A. (2018). The Amaryllidaceae Alkaloid Haemanthamine Binds the Eukaryotic Ribosome to Repress Cancer Cell Growth. Structure.

[73] Caron M.M.J., Emans P.J., Coolsen M.M.E., Voss L., Surtel D.A.M., Cremers A., van Rhijn L.W., Welting T.J.M. (2012). Redifferentiation of dedifferentiated human articular chondrocytes: Comparison of 2D and 3D cultures. Osteoarthr. Cartil..

[74] Housmans B.A.C., Neefjes M., Surtel D.A.M., Vitík M., Cremers A., van Rhijn L.W., van der Kraan P.M., van den Akker G.G.H., Welting T.J.M. (2022). Synovial fluid from end-stage osteoarthritis induces proliferation and fibrosis of articular chondrocytes via MAPK and RhoGTPase signaling. Osteoarthr. Cartil..

[75] Pichot F., Marchand V., Ayadi L., Bourguignon-Igel V., Helm M., Motorin Y. (2020). Holistic Optimization of Bioinformatic Analysis Pipeline for Detection and Quantification of 2′-O-Methylations in RNA by RiboMethSeq. Front. Genet..

[76] Rivera M.C., Maguire B., Lake J.A. (2015). Isolation of ribosomes and polysomes. Cold Spring Harb. Protoc..

[77] Penzo M., Rocchi L., Brugiere S., Carnicelli D., Onofrillo C., Couté Y., Brigotti M., Montanaro L. (2015). Human ribosomes from cells with reduced dyskerin levels are intrinsically altered in translation. FASEB J..

[78] Timur U.T., Jahr H., Anderson J., Green D.C., Emans P.J., Smagul A., van Rhijn L.W., Peffers M.J., Welting T.J.M. (2021). Identification of tissue-dependent proteins in knee OA synovial fluid. Osteoarthr. Cartil..

