# Supplementary material for: TGF-β2 Induces Ribosome Activity, Alters Ribosome Composition and Inhibits IRES-Mediated Translation in Chondrocytes
Source: Int J Mol Sci. 2024 May 5;25(9):5031. doi: 10.3390/ijms25095031 (PMC11084827; doi:10.3390/ijms25095031)
Supplement: Supplementary file 1 [file ijms-25-05031-s001.zip › ijms-2936717-supplementary.pdf]

Supplementary material

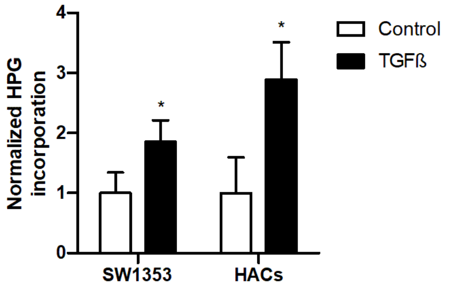

Supplementary Figure S1: HPG incorporation over a 24 hours period corresponds well with 30 minutes of ^{35S} methionine/cysteine incorporation. *Total protein translation was monitored over 24 hours by supplementation of media with the methionine analogue L-Homopropargylglycine (HPG) and normalized to DNA content per well. Bar graphs show Mean ± SD. SW1353 (n=4), HACs (n=8). * = p-value <0.05 in a t-test.*

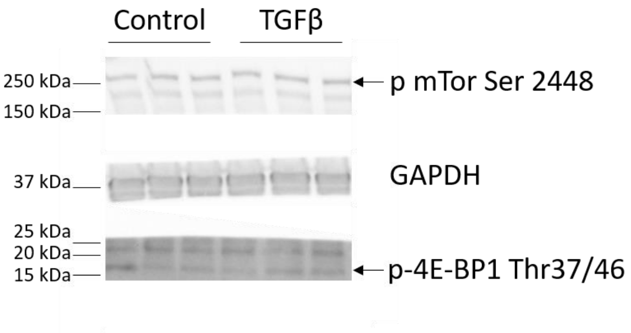

Supplementary Figure S2: TGF-β did not affect mTOR^{Ser2448} or 4E-BP1Thr$^{37/46}$ phosphorylation. *SW1353 were stimulated for 3 days with or without 10 ng/ml TGF-β and the medium was refreshed every 24 hours.*

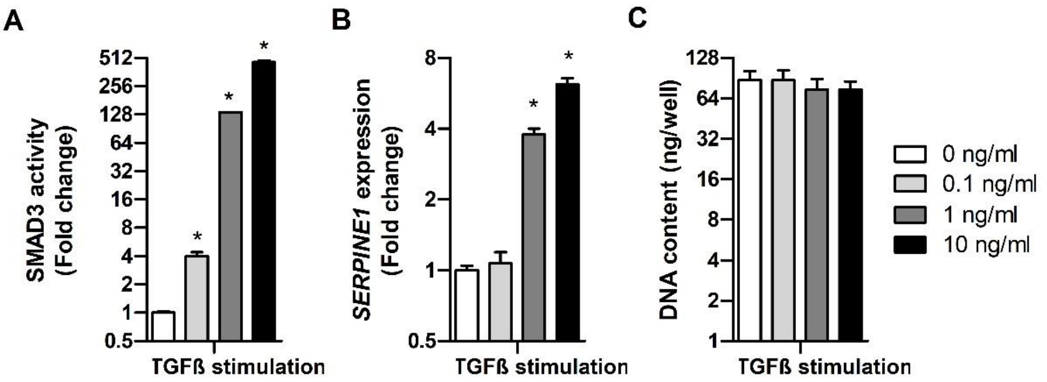

Supplementary Figure S3: Dose-response of TGF-β on SMAD3 transcriptional activity, SERPINE1 gene expression and DNA content. (A) *CAGA-luciferase assay at 6 hours post-stimulation (n=3).* ***(B)*** *SERPINE1 gene expression after 24 hours of stimulation (n=3).* ***(C)*** *DNA content as a function of TGF-β supplementation after 3 days of stimulation (n=12). Legend indicates the concentration of TGF-β.*

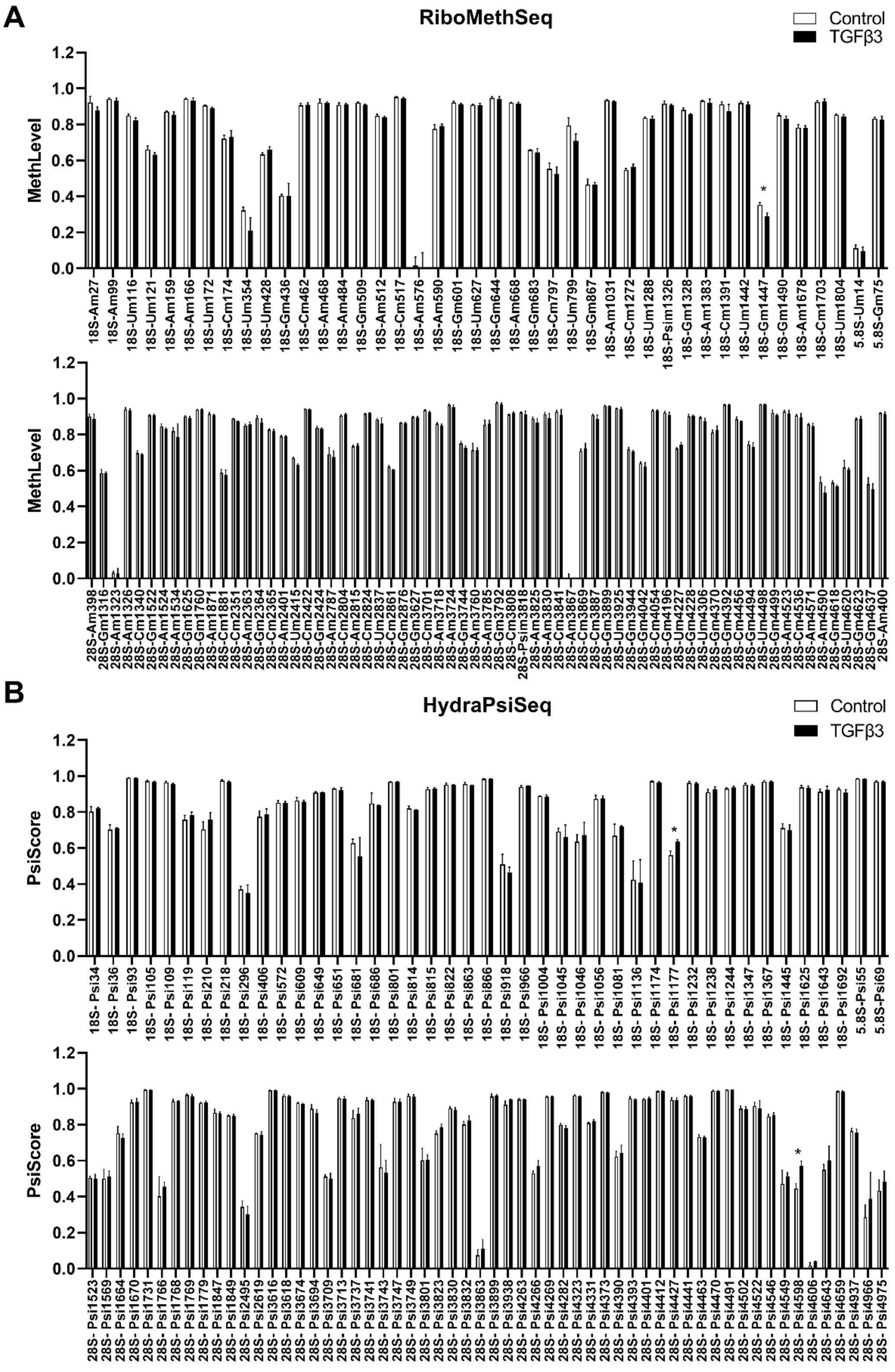

Supplementary Figure S4: 2'-O-methylation and pseudouridylation rRNA profiles after 3 days with or without TGF-β3. (A) *2'-O-methylation (RiboMethSeq) and* ***(B)*** *pseudouridylation (HydraPsiSeq) rRNA profiles. Top panels 5.8S rRNA and 18S rRNA, bottom panels 28S rRNA. Statistical analysis was done with a two-sided t-test. * = p-value <0.05 and ≥ 5% change.*

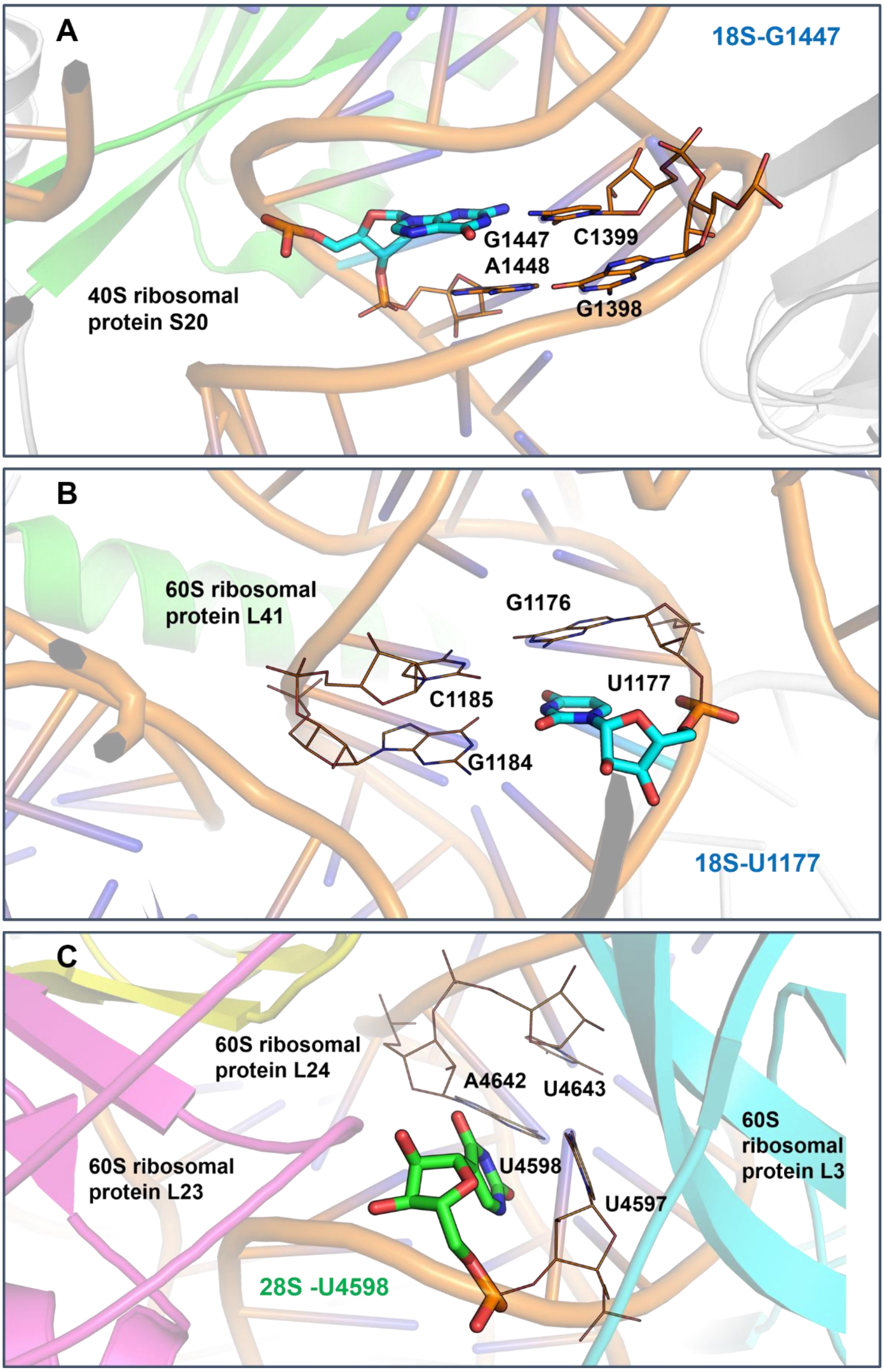

***Supplementary Figure S5: Close-ups of altered rRNA PTM locations in the human ribosome following TGF-β treatment. (A)** Gm1447 on 18S rRNA was decreased by TGF-β treatment and is located in close proximity of RPS20 (green). **(B)** ΨU1177 on 18S rRNA was increased by TGF-β treatment and located in the proximity of RPL41 (green). **(C)** ΨU4598 on 28S was increased by TGF-β treatment and located in the proximity of RPL3 (cyan), RPL23 (magenta) and RPL24 (yellow).*

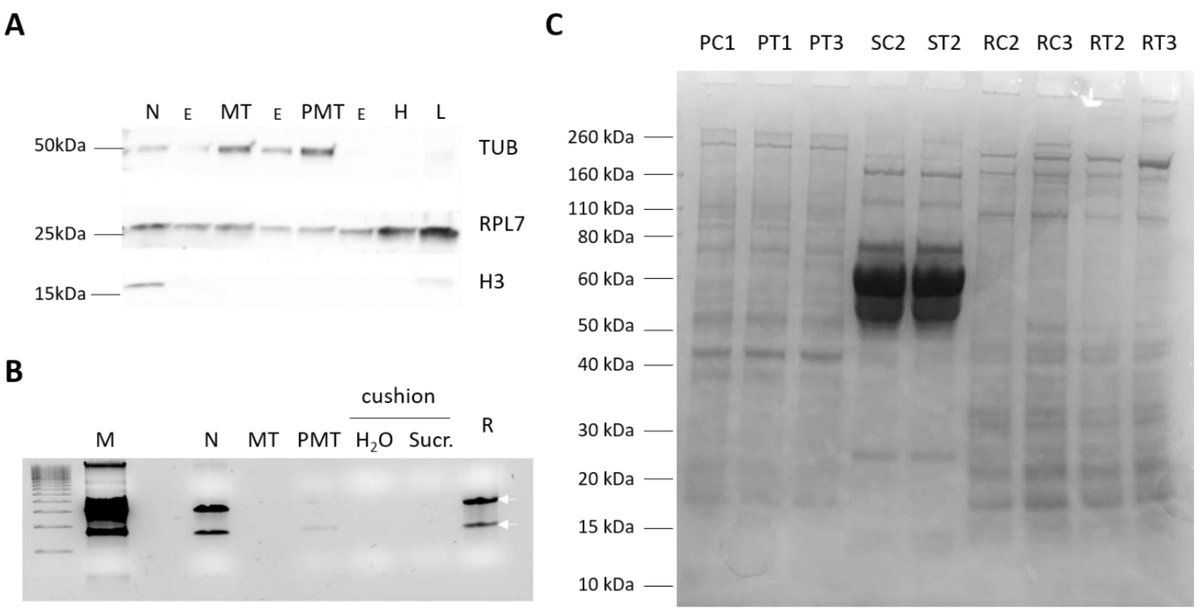

Supplementary Figure S6: Isolation of ribosomes using a sucrose cushion. *SW1353 cells were lysed with a Dounce homogenizer, nuclei (N) and mitochondria (MT) were removed by sequential centrifugation. The resulting post-mitochondrial fraction (PMT) was run over a sucrose cushion to obtain ribosomes (R).* ***(A)*** *Protein samples were generated from N, MT, PMT and Ribosomal fractions derived from either high salt washing (two cushions, one with 500 mM NaCl and 0.7 M Sucrose and the second with 70 mM NaCl, 1 M Sucrose) or low salt washing (two cushions of 0.7 M and 1M sucrose containing 25 mM NaCl). Samples were separated by SDS page, blotted to Nitrocellulose membranes and probed with anti-α-Tubulin (TUB), RPL7 or Histon 3 (H3) antibodies. E = empty well, some overflow between the slots is visible.* ***(B)*** *Total RNA isolates were separated by agarose gel electrophoresis.* H_2O *and Sucrose (Sucr.) are negative control samples taken from the top and bottom part of the cushion. White arrows indicate mature ribosomal RNAs, which are enriched in the ribosomes when compared to the PMT fraction. Note that nuclei contain a large amount of rRNA precursors and various processing intermediates.* ***(C)*** *Samples for LC-MS/MS (7.5 µg each) were run on a NuPAGE 4-12% Bis-Tris Protein Gel and stained with Coomassie blue. PC = proteome control, PT = proteome TGF-β, secretome control, ST = secretome TGF-β, RC = riboproteome control, RT = ribosomal proteome TGF-β.*
